# Ameliorative Effect of Daidzein on Cisplatin-Induced Nephrotoxicity in Mice via Modulation of Inflammation, Oxidative Stress, and Cell Death

**DOI:** 10.1155/2017/3140680

**Published:** 2017-08-02

**Authors:** Hongzhou Meng, Guanghou Fu, Jie Shen, Kezhen Shen, Zhijie Xu, Yiming Wang, Baiye Jin, Hao Pan

**Affiliations:** ^1^Department of Urology, the First Affiliated Hospital, College of Medicine, Zhejiang University, Hangzhou, Zhejiang, China; ^2^Key Laboratory of Combined Multi-Organ Transplantation, Ministry of Public Health, the First Affiliated Hospital, College of Medicine, Zhejiang University, Hangzhou, Zhejiang, China

## Abstract

Oxidative stress and inflammation are part and parcel of cisplatin-induced nephrotoxicity. The purpose of this work is to study the role of soy isoflavone constituent, daidzein, in cisplatin-induced renal damage. Cisplatin-induced nephrotoxicity was evident by the histological damage in proximal tubular cells and by the increase in serum neutrophil gelatinase-associated lipocalin (NGAL), blood urea nitrogen (BUN), creatinine, and urinary kidney injury molecule-1 (KIM-1). Cisplatin-induced cell death was shown by TUNEL staining and caspase-3/7 activity. Daidzin treatment reduced all kidney injury markers (NGAL, BUN, creatinine, and KIM-1) and attenuated cell death (apoptotic markers). In cisplatin-induced kidney injury, renal oxidative/nitrative stress was manifested by the increase in lipid peroxidation and protein nitration. Cisplatin induced the reactive oxygen species-generating enzyme NOX-2 and impaired antioxidant defense enzyme activities such as glutathione peroxidase (GPX) and superoxide dismutase (SOD) activities. Cisplatin-induced oxidative/nitrative stress was attenuated by daidzein treatment. Cisplatin induced CD11b-positive macrophages in kidneys and daidzein attenuated CD11b-positive cells. Daidzein attenuated cisplatin-induced inflammatory cytokines tumor necrosis factor *α* (TNF*α*), interleukin 10 (IL-10), interleukin 18 (IL-18), and monocyte chemoattractant protein-1 (MCP-1). Daidzein attenuated cell death *in vitro*. Our data suggested that daidzein attenuated cisplatin-induced kidney injury through the downregulation of oxidative/nitrative stress, immune cells, inflammatory cytokines, and apoptotic cell death, thus improving kidney regeneration.

## 1. Introduction

Cisplatin is a commonly used anticancer drug for the treatment of solid tumors. The mechanism of cancer cell killing is through its DNA-binding properties by forming adducts and stopping replication of cancer cells. One of the major side effects of cisplatin is nephrotoxicity and is mediated by preferential absorption of cisplatin in proximal tubules through a specific transporter [1]. Hydration has been used to alleviate this issue with some success. However, the dose-dependent nephrotoxicity is thus a limiting factor during cisplatin chemotherapy. The mechanism of cisplatin-mediated nephrotoxicity is mediated by apoptotic cell death induced by oxidative stress and inflammation.

Cisplatin is mainly excreted by kidneys, and high concentration of cisplatin accumulated there due to the basolateral organic cation system [2]. Cisplatin also accumulates in mitochondria and modulates its bioenergetics [3]. However, substantial literatures indicate that oxidative stress plays a critical role in renal damage [4]. Cell death associated with oxidative stress leads to inflammatory response and is highly relevant to the pathogenesis of cisplatin-induced nephrotoxicity [5].

Various natural bioactive compounds, which have antioxidant and anti-inflammatory properties, exhibit renoprotective activity in an animal model of cisplatin nephrotoxicity. Daidzein is found in soybeans and is a constituent of Chinese traditional medicine Nao Mai Tong formula. Daidzein is an isoflavone and has antioxidant, anti-inflammatory, and phytoestrogenic properties. Daidzein demonstrates anti-inflammatory effects on endotoxin-induced RAW 264.7 macrophages [6]. In a clinical trial, both soy and purified daidzein improved renal function [7]. Daidzein also inhibits STAT-1 and NF-*κ*B activations in an activated macrophage [8]. Daidzein has cardioprotective and antiarthritogenic effects on rheumatoid arthritis. A clinical trial (ClinicalTrials.gov NCT02075112) is currently ongoing with soy supplementation during cisplatin chemotherapy and radiation therapy for head and neck cancer to decrease side effects caused by treatments.

Here, we demonstrated that daidzein is protective against cisplatin-induced nephrotoxicity. The protective effect was mediated by its antioxidant and anti-inflammatory properties.

## 2. Materials and Methods

### 2.1. Mouse Experiments

All protocols were approved by the Committee on the Ethics of Animal Experiments of the First Affiliated Hospital, College of Medicine, Zhejiang University, under the guidance of the Chinese Academy of Sciences. The mouse strain C57BL/6 was used as described before [9]. Male mice of ~8 weeks of age with weights of 18–22 g were used in all experiments. Mice were sacrificed under deep anesthesia with 5% isoflurane followed by cervical dislocation on the third day (72 hours) after a single injection of cisplatin (cis-diammineplatinum (II) dichloride, Sigma) at dose 25 mg/kg i.p. in 5% DMSO/saline vehicle. High-quality daidzein (>98% pure) was purchased from Nanjing Zelang Medical Technology Co. Ltd. Daidzein was dissolved in DMSO/saline and administered at 200 mg/kg, i.p., for two days, starting 1 h after the cisplatin administration. Daidzein and vehicle were also administered alone (without cisplatin treatment) as a separate group.

### 2.2. Kidney Function

Serum levels of blood urea nitrogen (BUN) and creatinine were measured as described earlier [10]. Serum NGAL and urinary KIM-1 were measured from serum using Mouse NGAL Quantikine ELISA Kit and Mouse KIM-1 Quantikine ELISA Kit (R&D Systems China Co. Ltd., Changning, China) according to the manufacturer's instruction.

### 2.3. Histology

Periodic acid-Schiff (PAS) staining for histological examination was performed as described earlier [10]. Slides with PAS staining were examined based on the following four histological criteria and scored. Tubular damage in PAS-stained sections was examined under the microscope and scored based on the percentage of cortical tubules showing epithelial necrosis: 0—normal, 1—<10%, 2—10 to 25%, 3—26 to 75%, and 4—>75%. Tubular necrosis was defined as the loss of the proximal tubular brush border, blebbing of apical membranes, tubular epithelial cell detachment from the basement membrane, or intraluminal aggregation of cells and proteins. The morphometric examinations were performed in a blinded manner.

Protein nitrotyrosine staining using monoclonal anti-nitrotyrosine antibody (Cayman Chemical, NeoBioscience Technology, Shenzhen, China) was performed as described earlier [10].

### 2.4. Fluorescence Microscopy

Kidneys were sectioned with a microtome, deparaffinized, and stained as provided below with a fluorescence microscope. Apoptosis was detected in the kidneys by the TUNEL assay (Roche Diagnostics, Indianapolis, IN, USA) along with nuclear staining using Hoechst 33342 (Solarbio, China) as described earlier [10]. CD11b-conjugated FITC (BD Biosciences, USA) for neutrophils/monocytes (leukocytes) and nuclear stain Hoechst 33342 (Solarbio, China) were used in kidney sections.

### 2.5. Renal Apoptosis

Caspase-3/7 activity of the lysate was measured using Apo-ONE Homogenous Caspase-3/7 Assay Kit (Promega Corp., Madison, WI, USA) as described earlier [9]. Caspase-3/7 activity was presented as caspase-3 activity in the figure and text. The activity was expressed as fold change.

### 2.6. Renal HNE Protein Adducts and Protein Nitration

Nitrotyrosine content was evaluated by ELISA as described [9] HNE adducts were determined using OxiSelect™ HNE Adduct ELISA Kit (Cell Biolabs, Genetimes Technology Inc., Shanghai, China) as described earlier [9].

### 2.7. Quantitative Determination of SOD Activity

SOD activity was determined from tissue lysates using an SOD activity kit (Enzo Life Sciences International Inc., Plymouth Meeting, PA, USA) as described before [9].

### 2.8. Glutathione Peroxidase Assay

Glutathione peroxidase was measured using a Glutathione Peroxidase (GPX) Assay Kit (Abcam Trading Company Ltd., Shanghai, China) according to the manufacturer's instruction.

### 2.9. Glutathione Content

Glutathione (GSH) was determined by using a colorimetric kit (Jiancheng Bioengineering Institute, China) according to the manufacturer's instructions.

### 2.10. Real-Time PCR

Isolation of RNA and real-time PCR were carried out as described earlier [9, 10]. The primer sets for TNF*α* (PPM03113G), IL-18 (PPM03112B), IL-10 (PPM03017C), MCP-1 (PPM03151G), NOX2 (PPM32951A), and *β*-actin (PPM02945B) were purchased from Qiagen (Pudong, Shanghai, China).

### 2.11. Renal Western Blot

Western blot was performed as described previously [11].

### 2.12. Cell Culture and Flow Cytometry Analyses

HK-2 cells were grown and processed as described earlier [9]. Cisplatin was added at 50 *μ*M and vehicle or daidzein at 30 *μ*M after a 30 min delay to cisplatin addition for 24 hours. Flow cytometry experiments were performed as described earlier [9].

### 2.13. Statistical Analysis

All data were presented as the means ± SEMs. Multiple comparisons (Tukey) were performed using one-way ANOVA. The analyses were performed with GraphPad Prism software (GraphPad Software Inc., CA, USA). A *p* value <0.05 was considered statistically significant.

## 3. Results and Discussion

### 3.1. Effect of Daidzein on Cisplatin-Induced Tubular Damage, Kidney Injury, and Cell Death

**Figure 1:**
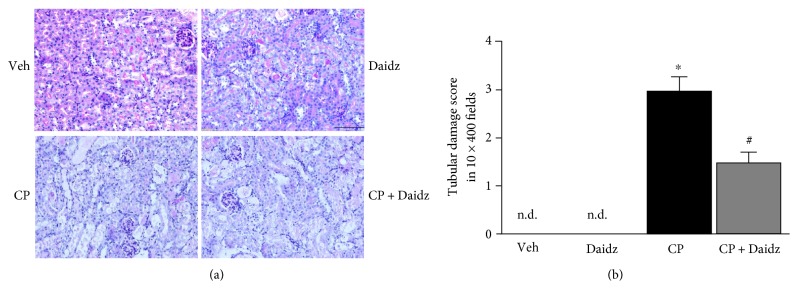
Effect of daidzein on cisplatin-induced kidney tubular damage in mice. (a) Cisplatin induced tubular damage as shown by PAS staining. The damage was attenuated by daidzein (daidz) treatment at dose 200 mg/kg. (b) Quantification of the tubular damage score from PAS-stained slide. Results are mean ± SEM (*n* = 6/group). ^∗^*p* < 0.05 versus vehicle and ^#^*p* < 0.05 versus cisplatin.

**Figure 2:**
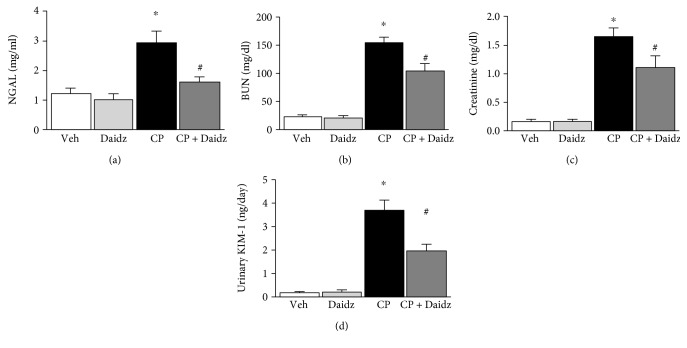
Effect of daidzein on cisplatin-induced renal dysfunction in mice. Cisplatin caused significant renal dysfunction as determined by the levels of NGAL (a), BUN (b), creatinine (c), and urinary KIM-1 at 72 hours (d). Cisplatin induced kidney injury which was attenuated by daidzein treatment. Results are mean ± SEM (*n* = 6/group). ^∗^*p* < 0.05 versus vehicle and ^#^*p* < 0.05 versus cisplatin.

**Figure 3:**
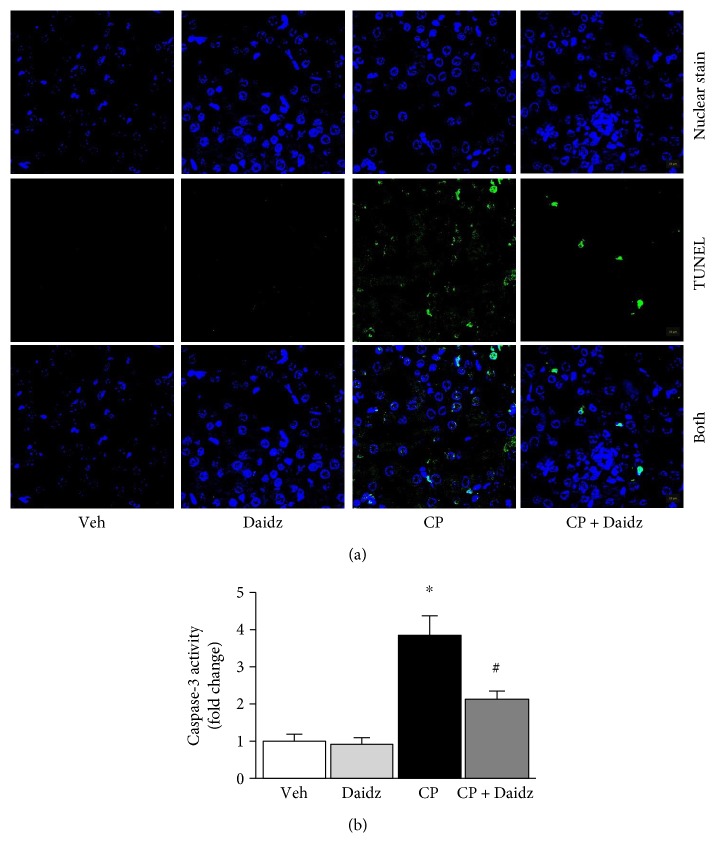
Effects of daidzein on cisplatin-induced cell death. Histological examination (a) demonstrated cisplatin-induced TUNEL staining (green) in the kidney and TUNEL staining was significantly attenuated with daidzein administration. Nuclei were stained with Hoechst 33342 (blue). (b) Caspase-3 activities were determined and daidzein attenuated cisplatin-induced caspase-3 activity. Results are mean ± SEM (*n* = 6/group). ^∗^*p* < 0.05 versus vehicle and ^#^*p* < 0.05 versus cisplatin.

Cisplatin administration to C57BL6 mice led to significant tubular damage at 72 hours as observed in PAS staining (Figure 1). Histological examination and quantification revealed vacuolation, protein cast formation, and desquamation of epithelial cells in the renal tubules. The damage was significantly attenuated by daidzein treatment in mice. Cisplatin also induced renal dysfunction as found by the kidney injury parameters such as NGAL, BUN, creatinine, and urinary KIM-1 (Figure 2). Cisplatin administration resulted in a 2.46-, 6.92-, 9.93-, and 20-fold increase in NGAL, BUN, creatinine, and KIM-1, respectively. Daidzein attenuated all kidney injury markers. Daidzein at 200 mg/kg reduced cisplatin-induced kidney injury as shown by a decrease in serum levels of NGAL (2.93 to 1.60), BUN (153.5 to 103.6), creatinine (1.65 to 1.11), and urinary KIM-1 (3.7 to 1.98). Cisplatin is known to cause apoptotic cell death in the kidney. We have observed a significant increase in TUNEL staining in cisplatin kidney, and the number reduced after daidzein treatment (Figure 3(a)). Quantitative determination of caspase-3/7 activity demonstrated that cisplatin induced 3.86-fold increases and daidzein treatment reduced 43.9% of caspase-3/7 activity (Figure 3(b)).

Nephrotoxic drug-related acute kidney injury in hospital was approximately 20% and increased to 66% for the elderly [5]. Cisplatin accumulated at high concentration in the kidneys by the renal transport system, and its toxicity is dose dependent [12]. Whole soy and its constituent daidzein have a positive effect on renal function in a clinical trial [7]. Pharmacokinetics of daidzein in human, mouse, and rat demonstrates its presence as glucuronides [13, 14]. Comparative pharmacokinetics of traditional Chinese medicine Nao Mai Tong in a rat study also demonstrates the presence of daidzein [15]. In our study, daidzein significantly reduced cisplatin-induced acute kidney injuries by improving kidney function and prevented tubular cell death.

### 3.2. Effect of Daidzein on Cisplatin-Induced Oxidative Stress and Impaired Antioxidant Defense

**Figure 4:**
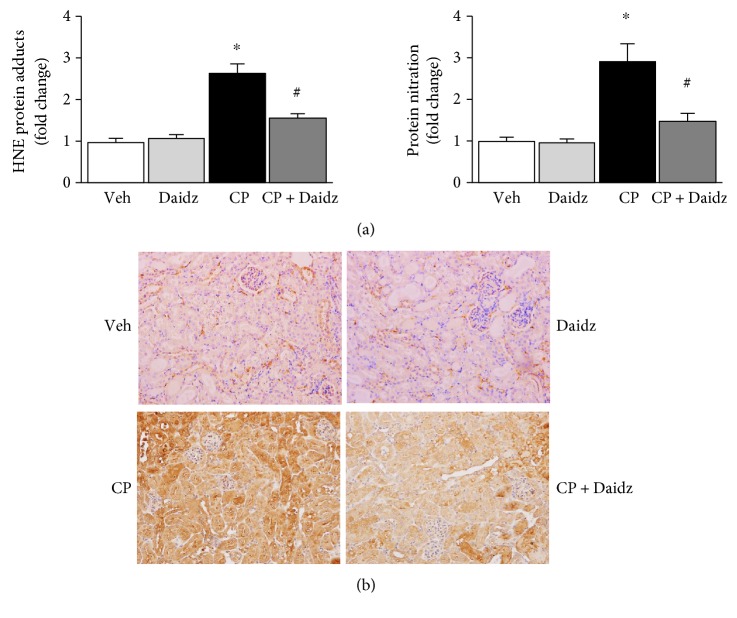
Effect of daidzein on cisplatin-induced oxidative/nitrative stress. (a) Quantitative measurement of HNE adducts and protein nitration by ELISA demonstrated cisplatin-induced lipid peroxidation and protein nitration. Daidzein attenuated both cisplatin-induced oxidative/nitrative stress markers. (b) Histological staining of protein nitration. A trend similar to quantitative protein nitration was observed. Results are mean ± SEM (*n* = 6/group). ^∗^*p* < 0.05 versus vehicle and ^#^*p* < 0.05 versus cisplatin.

Cisplatin-induced nephrotoxicity is mediated by oxidative stress [16]. We evaluated the effect of daidzein on cisplatin-induced oxidative footprints such as HNE protein adducts and protein nitration. Both oxidative stress markers HNE protein adducts and protein nitration were increased 2.6- and 2.9-fold in cisplatin-treated mice (Figure 4(a)). Treatment with daidzein reduced 39.9% and 48.7% of HNE protein adducts and protein nitration, respectively. We also examined protein nitration by histological staining, and daidzein significantly reduced cisplatin-induced protein nitration (Figure 4(b)). In all above experiments, daidzein did not change any oxidative stress marker when administered alone.

**Figure 5:**
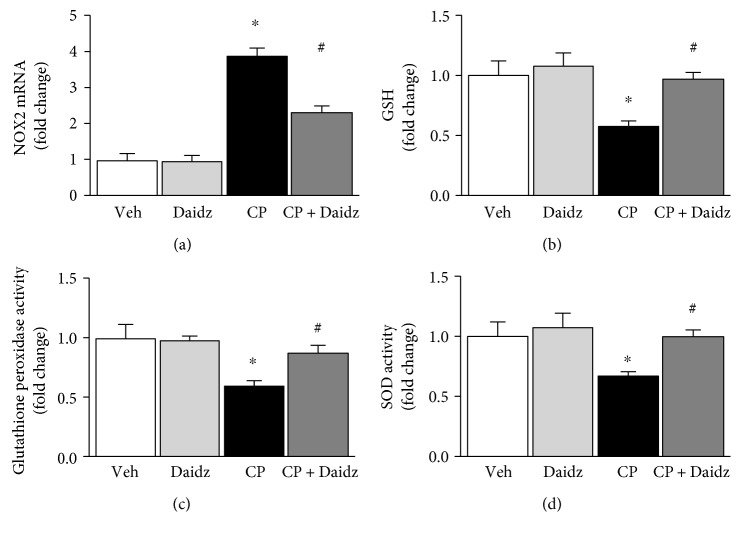
Effect of daidzein on cisplatin-induced changes in the ROS-generating enzyme NOX2 and antioxidant defense in mice. (a) Cisplatin induced the ROS-generating enzyme NOX2 mRNA as determined by real-time PCR, and daidzein attenuated cisplatin-reduced reduced glutathione reserve, glutathione peroxidase activity, and SOD activity. Daidzein administration restored those antioxidant defenses close to the control group. Results are mean ± SEM (*n* = 6/group). ^∗^*p* < 0.05 versus vehicle and ^#^*p* < 0.05 versus cisplatin.

The balance of reactive oxygen species- (ROS-) generating enzymes and antioxidant defense enzymes is critical for cisplatin nephrotoxicity [10, 17]. We found that cisplatin-induced gene expression of the ROS-generating enzyme NOX2 is significantly attenuated by daidzein (3.8- to 2.3-fold, Figure 5(a)). Glutathione plays a critical role in cisplatin-induced kidney injury [18]. Daidzein also improved up to 66% of cisplatin-mediated depletion of reduced glutathione in mouse kidney (Figure 5(b)). In addition to that, daidzein enhanced a cisplatin-mediated reduction in glutathione peroxidase activity and total SOD activity up to 49% and 55%, respectively (Figures 5(c) and 5(d)).

Oxidative stress plays a critical role in cisplatin-induced acute kidney injury [16, 19, 20]. Previous studies show that cisplatin mediated an increase in lipid peroxidation (one oxidative stress marker) and is attenuated by flavonoids and antioxidants [21–23]. Protein nitration is mediated by peroxynitrite and another hallmark of cisplatin-induced oxidative stress in the kidney [9]. Here, we demonstrated that daidzein attenuated cisplatin-induced protein nitration and lipid peroxidation (HNE adducts). NOX2 is one among several sources for oxidative stress [24, 25]. Daidzein attenuated cisplatin-induced NOX2 expression. Antioxidant defense is also critical in cisplatin-induced nephropathy [9, 26]. Cisplatin reduces the reserve of reduced glutathione, SOD activity, and glutathione peroxidase activity in kidneys [27–29]. Consistent with earlier studies, cisplatin impaired reduced glutathione reserve and decreased glutathione peroxidase activity and SOD activity. Daidzein improved all three antioxidant defense mechanisms and thus ameliorated cisplatin-induced oxidative stress.

### 3.3. Effect of Daidzein on Cisplatin-Induced Leukocyte Infiltration and Inflammatory Cytokines in the Kidney

**Figure 6:**
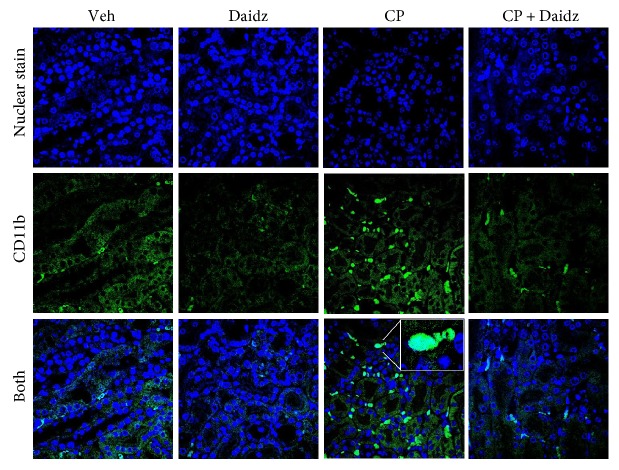
Effect of daidzein on cisplatin-induced CD11b-positive monocyte/macrophage in mice. Immunofluorescence examination revealed significant CD11b-positive cells (yellow) of the cisplatin-treated group. Nuclear staining (blue) was carried out using Hoechst 33342. In the cisplatin group, a zoom image of single cells was provided as an inset to demonstrate that staining covers surface staining and a larger area than nuclear staining. Daidzein treatment reduced the number of CD11b-positive cells. Either vehicle (Veh) or daidzein (daidz) control group does not have any CD11b-positive cells.

**Figure 7:**
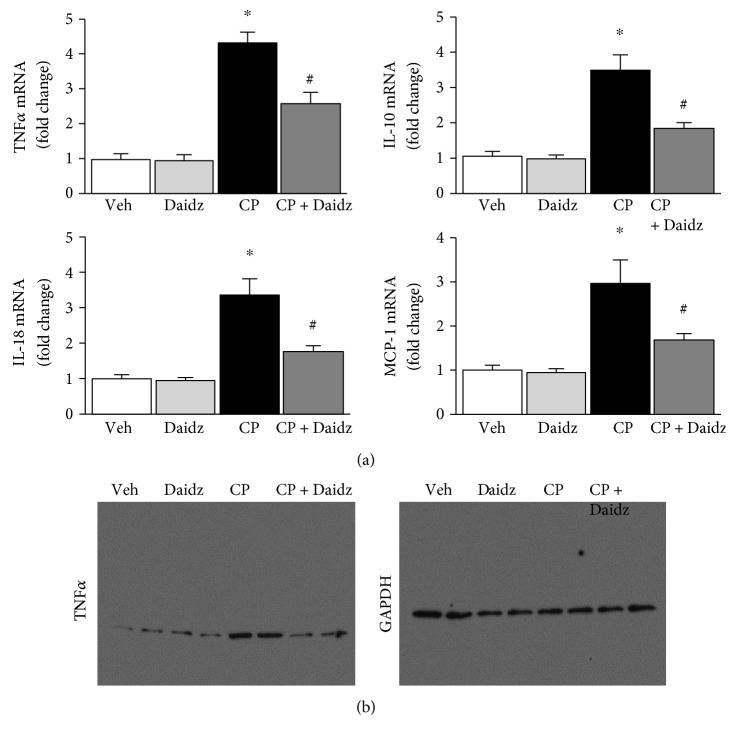
Effect of daidzein on cisplatin-induced proinflammatory cytokines in mice. (a) Real-time PCR-based analyses of proinflammatory cytokines TNF*α*, IL-10, IL-18, and MCP-1 indicated a profound increase in cisplatin-treated mice. Daidzein treatment attenuated cisplatin-induced cytokine MRNA expression. Results are mean ± SEM (*n* = 6/group). ^∗^*p* < 0.05 versus vehicle and ^#^*p* < 0.05 versus cisplatin. (b) Western blot analyses of TNF*α* and control GAPDH.

Cisplatin-induced inflammatory response followed by infiltration of neutrophils and macrophages has been reported earlier [10, 19, 24]. Consistent with earlier findings, we observed CD11b-positive cells in cisplatin-induced kidney injury and daidzein significantly reduced CD11b-positive cells (Figure 6). Cisplatin induces several cytokines such as TNF*α*, IL-10, IL-18, and MCP-1 in kidney injury [30–32]. Cisplatin induced TNF*α*, IL-10, IL-18, and MCP-1 mRNA expression to 4.3-, 3.5-, 3.4-, and 2.98-fold, respectively (Figure 7(a)). Daidzein treatment attenuated 39.9%, 46.2%, 47%, and 43.2% of TNF*α*, IL-10, IL-18, and MCP-1 mRNA expression, respectively. We further verified one of the cytokines TNF*α* by Western blot analyses (Figure 7(b)), and the result was consistent with mRNA level. GAPDH was used as a loading control.

In the pathogenesis of cisplatin-induced nephrotoxicity, inflammation plays another major role [12, 33]. Different immune cells, namely, neutrophils, macrophages, T cells, and dendritic cells, play their role during the inflammatory response [12]. Neutrophils and monocyte-derived macrophages are myeloid cells which pursue common goals to neutralize danger in cisplatin-mediated nephropathy [32, 34]. CD11b is a *α*M integrin, a member of the integrin family, primarily expressed on monocytes/macrophages. Consistent with earlier data, we also observed a significant increase in a CD11b-positive macrophage in cisplatin-induced kidney injury [35]. Daidzein reduced cisplatin-induced macrophage accumulation in the kidneys. Daidzein inhibits production of nitric oxide and IL-6 in a lipopolysaccharide-induced macrophage [36].

Cisplatin activates the NF-*κ*B pathway, thus facilitating inflammatory cytokines such as TNF*α*. Daidzein attenuated cisplatin-induced TNF*α*, and consistent with previous findings, berberine, curcumin, and chlorogenic acid mediated a renoprotective effect [21, 37, 38]. IL-18 is also crucial in cisplatin-mediated toxicity [39]. Genetic deletion of caspase-1, which cleaved IL-18 to make it active, reduced cisplatin kidney injury and neutrophil infiltration [40]. IL-18 was induced by cisplatin and attenuated with daidzein treatment in our study. IL-10 and MCP-1 also play a role in cisplatin-induced kidney injury [31, 41, 42]. We also observed that cisplatin induced both IL-10 and MCP-1 mRNA and those were attenuated by daidzein. Dendritic cells produce IL-10, and the modulation of dendritic cells by daidzein in cisplatin nephropathy cannot be excluded.

### 3.4. Effect of Daidzein on Cisplatin-Induced Cell Death of Proximal Tubular Cell Line *In Vitro*

**Figure 8:**
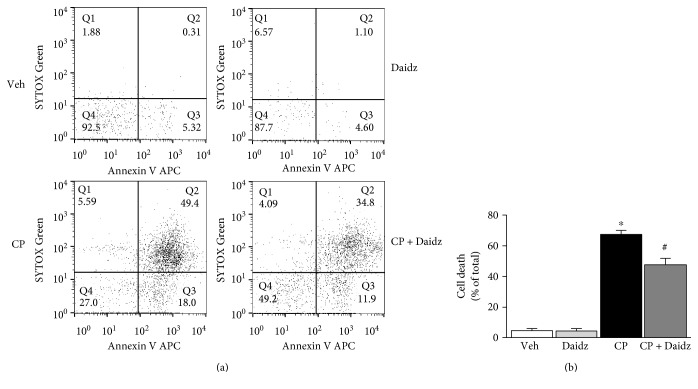
Effect of daidzein on cisplatin-induced cell death *in vitro*. (a) Representative dot plot of flow cytometric data of an HK-2 cell treated with either saline or daidzein in the presence or absence of cisplatin. *x*-axis represented the apoptotic cell death marker Annexin V whereas *y*-axis represented the dead cell marker SYTOX Green. (b) Quantitative determination of cell death (combined Q2 and Q3) among different groups. Results are mean ± SEM (*n* = 3/group). ^∗^*p* < 0.05 versus vehicle and ^#^*p* < 0.05 versus cisplatin.

Daidzein reduced both oxidative stress and inflammation. We also examined its effect on cell death under *in vitro* condition using HK-2 proximal tubular cell line. Cisplatin induced both apoptotic and necrotic cell death at 50 *μ*M for 24 hours (Figure 8(a)). Daidzein addition at 30 *μ*M attenuated cell death by 28.29% (Figure 8(b)). There was no effect of daidzein on HK-2 cell death when added without cisplatin.

Daidzein-mediated protection of cisplatin-induced proximal tubular cell death demonstrated its direct role. Cisplatin-induced nephrotoxicity is a far more complex situation where interplay of oxidative stress, inflammation, and cell death is partially correlated. Because anti-inflammatory properties of daidzein is reported earlier and our data demonstrated its direct role in inflammatory cytokines, we concluded that daidzein had anti-inflammatory properties in addition to its function to prevent cell death. It is also important to note that daidzein has antiestrogenic properties [43]. However, these specific properties of daidzein had no effect on our study as we have used male mice.

## 4. Conclusions

**Figure 9:**
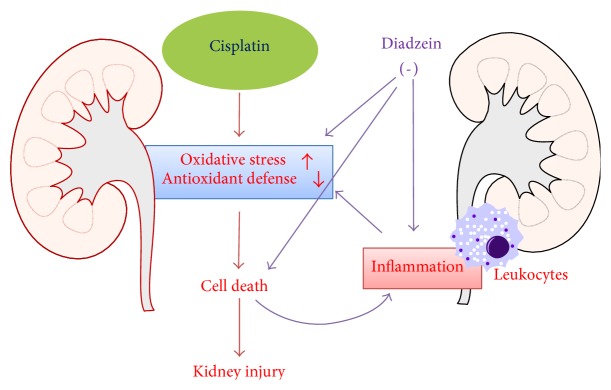
Schematic diagram of the protection mechanism of daidzein in cisplatin-induced kidney injury. EGCG inhibit cisplatin-induced ROS by attenuating ROS-generating enzymes and improving cisplatin-impaired antioxidant defense mechanisms in the renal tubular cells which caused cell death. Cell death also leads to proinflammatory response with cytokines and infiltrating leukocytes with the additional release of ROS. Daidzein attenuates cell death directly. Daidzein also neutralize cytokines and infiltrating leukocytes. Both antioxidant and anti-inflammatory effects leads to reduced cell death, thus protecting against cisplatin-induced kidney injury.

Cisplatin-induced tubular cell damage, cell death, and associated kidney injury were significantly attenuated with the administration of daidzein in mice. The molecular mechanism of protection is mediated through the interplay of oxidative stress and inflammation (Figure 9). Daidzein modulated cisplatin-induced lipid peroxidation, protein nitration, and NOX2 mRNA. Daidzein also improved cisplatin-impaired antioxidant defense such as reduced glutathione reserve, GPX activity, and SOD activity. Cisplatin-induced macrophage accumulation and proinflammatory cytokine production were attenuated by daidzein.
